# Supplementary material for: Tribological Properties of Nitrate Graphite Foils
Source: Nanomaterials (Basel). 2024 Sep 15;14(18):1499. doi: 10.3390/nano14181499 (PMC11434555; doi:10.3390/nano14181499)
Supplement: Supplementary file 1 [file nanomaterials-14-01499-s001.zip › X-ray data/macrostress/01Hz/160-200/dens_13/TD/188.doc]

**Analysis Results**

**General Information**

| Analysis date | 2024/05/15 09:55:18 | | |
| --- | --- | --- | --- |
| Sample name | 01_160_13_RD | Measurement date | 2024/05/14 12:53:08 |
| File name | 188.raw | Operator | Administrator |
| Comment | Psi = 0.000 | | |

**Measurement profile**

**Measurement conditions**

| X-Ray | 40 kV , 30 mA | Scan speed / Duration time | 1.0000 sec |
| --- | --- | --- | --- |
| Goniometer |  | Step width | 0.0200 |
| Attachment | - | Scan axis | 2theta/theta |
| Filter | K-beta filter | Scan range | 84.0000 - 90.0000 |
| CBO selection slit | BB | Incident slit | 2/3?‹\|2/3deg. |
| Diffrected beam mono. |  | Length limiting slit | - |
| Detector | Scintillation counter | Receiving slit #1 | Open |
| Scan mode | STEP | Receiving slit #2 | 0.30mm |

**Qualitative analysis results**

| Phase name | Formula | Figure of merit | Phase reg. detail | DB card number |
| --- | --- | --- | --- | --- |

| Phase name | Formula | Space group | Phase reg. detail | DB card number |
| --- | --- | --- | --- | --- |

**Peak list**

| No. | 2-theta(deg) | d(ang.) | Height(cps) | FWHM(deg) | Int. I(cps deg) | Size(ang.) | Phase name |
| --- | --- | --- | --- | --- | --- | --- | --- |
| 1 | 86.956(9) | 1.11947(9) | 2528(50) | 0.653(11) | 2641(35) | 171(3) | Unknown |
| 2 | 86.942(10) | 1.11962(10) | 2218(47) | 0.681(11) | 2386(16) | 164(3) | Unknown |
| 3 | 86.939(14) | 1.11965(14) | 1479(38) | 0.777(12) | 1728(38) | 144(2) | Unknown |
| 4 | 86.906(18) | 1.11999(18) | 842(29) | 0.899(15) | 1094(19) | 124(2) | Unknown |
| 5 | 86.91(3) | 1.1200(3) | 416(20) | 0.98(2) | 562(22) | 114(3) | Unknown |
| 6 | 86.96(7) | 1.1194(7) | 167(13) | 1.15(5) | 225(29) | 98(4) | Unknown |

**Parameters used for WPPF**

Profile parameters

| Common parameter | Background | Data | 188-1 |
| --- | --- | --- | --- |
|  |  | Function name | B-spline |
|  |  | param0 | 905.69410918384824 |
|  |  | param1 | 911.98270310545206 |
|  |  | param2 | 893.70991035071052 |
|  |  | node0 | 84 |
|  |  | node1 | 90 |
| Common parameter | Peak shift |  |  |
|  |  | Function name | Shift axial displacement |
|  |  | param0 | 0 |
|  |  | param1 | 0 |
|  |  | param2 | 0 |

Structure parameters

| Data set name | Phase Name | Element | x | y | z | Occupancy | Temperature factor |
| --- | --- | --- | --- | --- | --- | --- | --- |

| Data set name | Rwp | Rp | Re | S | Chi^2 | Maximum shift/e.s.d. |
| --- | --- | --- | --- | --- | --- | --- |
| 188-1 | 0 | 0 | 0 | 0 | 0 | 0 |
| 188-2 | 0 | 0 | 0 | 0 | 0 | 0 |
| 188-3 | 0 | 0 | 0 | 0 | 0 | 0 |
| 188-4 | 0 | 0 | 0 | 0 | 0 | 0 |
| 188-5 | 0 | 0 | 0 | 0 | 0 | 0 |
| 188-6 | 0 | 0 | 0 | 0 | 0 | 0 |
| 188-7 | 0 | 0 | 0 | 0 | 0 | 0 |

**Lattice constants**

Angular correction

Analysis results

| Data set name | a(A) | b(A) | c(A) | alpha(deg) | beta(deg) | gamma(deg) |
| --- | --- | --- | --- | --- | --- | --- |

| Phase name | a(A) | b(A) | c(A) | alpha(deg) | beta(deg) | gamma(deg) | V(A^3) |
| --- | --- | --- | --- | --- | --- | --- | --- |

LCGraph.emf

**Crystallinity**

| Data set name | Crystallinity(%) |
| --- | --- |

CrystallinityGraph.emf

**Crystallite size and lattice strain**

Williamson-Hall method

| Data set name | Crystallite size(A) | Strain(%) |
| --- | --- | --- |

| Phase name | Crystallite size(A) | Distribution RSD | Strain(%) | Distribution type |
| --- | --- | --- | --- | --- |

CSSGraph.emf

**Quantitative analysis results (RIR)**

RIRGraph.emf

**Quantitative analysis results (WPPF)**

**Quantitative analysis results (standard addition method)**

Calibration data

QuantityCalibration.emf

**Quantitative analysis results (External Standard method)**

Calibration data

QuantityCalibration.emf

**Quantitative analysis results (internal standard method)**

Calibration Data

QuantityCalibration.emf

**Stress**

Stress constants

| Phase name | Unknown | Young's modulus(MPa) | 149500 |
| --- | --- | --- | --- |
| Stress-free 2-theta angle(deg) | 87.023 | Poisson's ratio | 0.300 |
| Wavelength(ang) | CuKa (1.541836) | Stress constant(MPa/deg) | -1057.11100 |

Analytical conditions

|  | Psi angle(deg) | (sin(psi))^2 | 2-theta(deg) |
| --- | --- | --- | --- |
| * | 0 | 0 | 86.956(9) |
| * | 5 | 0.008 | 86.942(10) |
| * | 10 | 0.03 | 86.939(14) |
| * | 15 | 0.067 | 86.906(18) |
| * | 20 | 0.117 | 86.91(3) |
|  | 25 | 0.179 | 86.96(7) |

Analysis results

|  | Stress +/-Reliability(MPa) | Fomula | 2-theta(Psi=0)(deg) |
| --- | --- | --- | --- |
| + | 558 +/- 167 | Y=-0.53X+86.952 | 86.956(9) |
| - | - | Y=-0.53X+86.952 |  |

**Cluster analysis results**

Dendrogram

ClusterDendrogram.emf

Measurement profiles

Cluster

Sample well

ClusterSamplePlate.emf

PCA view

ClusterPCA3DGraph.emf

Eigenvalue graph

ClusterPCAGraph.emf

**Crystallite size distribution analysis results**

Crystallite size distribution

Crystallite size distribution graph

**Crystal structure analysis results**

Indexing

| Phase name | Formula | Figure of merit | Phase reg. detail | DB card number |
| --- | --- | --- | --- | --- |

Quantitative analysis results

Lattice information

| Phase name | a(A) | b(A) | c(A) | alpha(deg) | beta(deg) | gamma(deg) | V(A^3) |
| --- | --- | --- | --- | --- | --- | --- | --- |

| Phase name | Space group | Z | Z' | Calc. density(g/cm^3) |
| --- | --- | --- | --- | --- |

Structure determination

Refinement

Measurement range: 84.0000-90.0000deg Refinement range: 84.0000-90.0000deg (1.09 A)

Number of refined parameters: 0

| Phase name | Atomic coords | # of indep. reflns |
| --- | --- | --- |

Rwp = -	S = -

Crystal structure

CrystalGraph.emf
